# Single nucleotide polymorphism at alcohol dehydrogenase-1B is associated with risk of esophageal squamous cell carcinoma

**DOI:** 10.1186/1475-2867-14-12

**Published:** 2014-01-31

**Authors:** Bo Ye, Chun-Yu Ji, Yi Zhao, Wang Li, Jian Feng, Xu Zhang

**Affiliations:** 1 Department of Thoracic Surgery, Shanghai Chest Hospital, Shanghai Jiaotong University, Huaihaixi Road 241, Shanghai 200030, P.R China; 2 Department of Oncology, The First Affiliated Hospital of Dalian Medical University, Zhongshan Road 222, Dalian, Liaoning 116011, China; 3 Renji-MedX Clinical Stem Cell Research Center, Renji Hospital, Shanghai Jiao Tong University School of Medicine, Shanghai 200127, P.R China; 4 Department of Thoracic Surgery, The Second Affiliated Hospital of Dalian Medical University, Zhongshan Road 467, Dalian, Liaoning 116023, China

**Keywords:** Single nucleotide polymorphism, Alcohol dehydrogenase-1B, Esophageal squamous cell carcinoma

## Abstract

**Background:**

Esophageal squamous incidence in many developed countries has increased dramatically over last decades, while the underlying mechanism of the biogenesis of ES was still unknown.

**Methods:**

Here, we investigate 1001 subjects with esophageal cancer recruited from the affiliated hospital of Shanghai Jiao Tong University from Jan. 1, 2001 to Feb. 2, 2004. Single nucleotide polymorphism (SNP) of alcohol dehydrogenase-1B (ADH1B) was performed, and the recombinant plasimd containing ADH1B was constructed. Then, the ADH1B was purified and the enzymatic activity was assayed according to the methodology of Quayle. Furthermore, the effect of ADH1B on proliferation of human esophageal squamous cell lines was determined and the underlying mechanism of ADH1B was investigated.

**Results:**

Logistic regression analyses revealed that subjects carrying the GG variant homozygote had a significant 2.81-fold (adjusted OR = 2.81; 95% CI = 2.18-3.62) increased risk of esophageal cancer. We found that SNP of ADH1B (*GG*) significantly promotes cell proliferation in ESGG. ADH1B (*GG*) could down-regulate endogenous ADH1B expression at posttranscriptional level. Moreover, re-expression of ADH1B in cells transfected with ADH1B (*AA*) significantly inhibits cell proliferation.

**Conclusions:**

Our data implied that ADH1B (*GG*) could promote cell proliferation in human ESGG through regulating the enzyme activity of ADH1B. Therefore, we propose that ADH1B might be used as a therapeutic agent for human ESGG.

## Introduction

China was one of the countries with highest esophagus cancer risk in the world [1]. A rapid increase in incidence and mortality from esophageal adenocarcinoma (EA) has been observed over the past four decades in China [2]. Factors in the pathogenesis of esophageal cancer can be divided into the external cause and internal cause: external cause is mainly refers to the external stimulating factors [3] and internal cause is mainly refers to the patient’s genetic factors [4]. Epidemiological studies indicate that use of tobacco and consumption of alcohol are major risk factors for esophageal cancer [5]. However, only a subset of individuals exposed to tobacco and alcohol develop esophageal cancer, suggesting a role of host susceptibility factors in cancer development. Some studies have suggested that genetic polymorphisms might explain individual differences in susceptibility to esophageal cancer [6]. Esophageal cancer is often occurred under the continuous effect of exogenous factors on internal factors.

Some subtypes of cancer are linked to particular risk factors, such as age, sex, heredity and tobacco smoking and heavy alcohol usage. Alcohol intake may be causally related to cancer of the oral cavity, pharynx, larynx and esophagus [7]. Ethanol is oxidized to acetaldehyde and then to acetate by alcohol dehydrogenase (ADH) and aldehyde dehydrogenase (ALDH); both of which have genetic polymorphisms [8]. The genetic polymorphisms of alcohol-metabolizing enzymes modulate individual differences in alcohol-oxidizing capability and drinking behavior [9]. In humans, the major enzymes involved in the alcohol‑metabolizing pathways are alcohol dehydrogenase 1B (ADH1B) and aldehyde dehydrogenase 2 (ALDH2). Alcohol is first oxidized by ADH to acetaldehyde, which is oxidized to acetate by ALDH. These enzymes are mainly expressed in the liver, but are also present in the gastrointestinal tract [10]. The gene encoding the ADH1B enzyme is located on chromosome 4q22, and the ADH1B gene (encoding for subunit β) is the locus responsible for the majority of the ADH activity on ethanol in the liver [11]. ADH1B is a low Km (Michaelis constant)-class enzyme and exhibits high activity in catalyzing ethanol to acetaldehyde [12]. The most frequently reported locus is ADH1B Arg47His (rs1229984). In ADH1B His/His individuals, which are associated with flushing or other reactions to alcohol, the activity of ADH1B has been demonstrated to be decreased by 40-fold [13].

A recent genome-wide association study identified the variation of ADH1B rs1229984 and ALDH2 rs671 polymorphisms as risk factors for esophageal cancer [14]. Another genome-wide association study reported that variations of ADH1B rs1229984 and ALDH2 rs671 coupled with alcohol drinking and smoking synergistically enhanced the risk of esophageal cancer [15]. We evaluated the association between ADH1B genotypes and susceptibility to esophageal cancer in a hospital-based case–control study. Genotyping analyses were conducted for the two SNPs with 1001 ESGG cases and 1001 controls in a Chinese population. Here, we provided evidence that ADH1B rs1229984 GG is one of the risks to initiate the Human esophageal cancer. Ectopic expression of ADH1B was able to significantly inhibit esophageal cancer *in vitro and in vivo* by increasing the consuming of alcohol. Furthermore, we sought to investigate the associations between measures of ADH1B and risk of progression to esophageal cancer in gene, protein and cellular level. This study provide the specific therapeutic target genes for targeting therapy in Human esophageal cancer. However, further experiments are still needed to confirm our results.

## Material and methods

### Ethical approval of the study protocol

This hospital-based case–control study was sved by the Ethic Committee of Shanghai Chest Hospital Affiliated to Shanghai Jiao Tong University. All participating subjects provided written informed consent prior to inclusion in the study.

### Study subjects

A total of 1001 subjects with esophageal cancer were consecutively recruited from the affiliated hospital of Shanghai Jiao Tong University between Jan. 1, 2001 and Feb. 2, 2004. All cases of esophageal cancer were diagnosed as ESGG by pathological means. The exclusion criteria were patients who previously had cancer, any metastasized cancer, radiotherapy or chemotherapy. The controls were patients without cancer who were frequency-matched to the cases with regard to age and gender, were recruited from the two above mentioned hospitals during the same period. The majority of control subjects had trauma or infectious diseases. Each subject was personally questioned by trained interviewers using a pre-tested questionnaire to obtain information on demographic data (e.g., age, gender) and related risk factors (including tobacco smoking and alcohol consumption). Following the interview, 2 ml samples of venous blood were collected from each subject. Individuals who smoked one cigarette per day for >1 year were defined as ‘smokers’. Subjects who consumed ≥3 alcoholic drinks a week for 6 months were considered to be ‘alcohol drinkers’.

### Isolation of DNA and genotyping of ADH1B

Blood samples were collected from patients using Vacutainers and transferred to tubes lined with ethylene diamine tetra-acetic acid (EDTA). Genomic DNA was isolated from whole blood using the QIAamp DNA blood mini kit (Qiagen, Berlin, Germany). Genotyping was conducted of ADH1B gene was performed using a PCR-RFLP assay on a Gene Amp PCR System 9700 (Applied Bio-systems). The amplification conditions for gene was as follows: an initial denaturing step of 95°C for 5 min, followed by 40 cycles of 95°C for 10 s, 60°C for 30 s, and 74°C for 45 s and a final elongation step of 74°C for 2 min. PCR primers were designed for SNPs of the ADH1B genes. ADH1B-specific allele was amplified with primer set (ADH1B_RFLP_F: 5′-CCTTGGGGATAAACTGAATCTT-3′; ADH1B_RFLP_R: 5′-GAAATCCTGGATGGTGAACC-3′; amplicon 348 bp). The reaction mixture contained 7.5 ul of distilled water, 12.5 ul of 2X PCR buffer for KOD plus, 2.5 ul of 2 mM dNTPs, 10 pmol each primers for ADH1B, and 0.5 U of KOD plus polymerase.

### Construction of recombinant plasmid containing ADH1B

Human ADH1B cDNA located between EcoR I and Not I site of pUCm-T was cloned. According to the information about SNPs in the human ADH1B gene, point mutations were introduced into the wild-type ADH1B using QuickChange site-directed mutagenesis kit (Stratagene). We have generated the following three kinds of missense mutations reported previously: AA, GG and AG. The introduction of the mutations was verified by full sequencing. The ADH1B cDNA was sub-cloned into the EcoR I and Not I site of pEASY-T1, resulting in the production of pEASY-T1-ADH1B. pEASY-T1-ADH1B has EcoR I and Not I sites upstream and downstream of the ADH1B expression cassette, respectively. The EcoR I/Not I digested fragments of pEASY-T1-ADH1B were ligated with pPICZB, resulting in pPICZB-ADH1B. The recombined plasmid was transfected by electroporation of *Pichia pastoris.*

### Enzyme purification and assays

Cells were harvested by centrifugation and washed with 50 mM Tris–HCl, pH8.0 (buffer A). The cell paste was frozen at −20°C until further use. The cells were disrupted with single passage through a French press operated at 20,000 lb/in^2^. The cellular debris was removed centrifugation at 8, 000 × g for 30 min. Protein ADH1B in the supernatant was purified by DEAE-Sepharose. ADH1B activity was measured as the increase in A340 according to the method of *Quayle*[16], using 0.025% Triton X-100-treated tuber or soluble proteins that were extracted as previously described. 1) Add the ADH enzyme to each solution; 2) stop the ADH activity and create a color reaction that indicates the alcohol that has been broken down; 3) measure the amount of color produced in each tube using a spectrophotometer; 4) graph the results by plotting the absorbance obtained for the alcohol solutions.

### Cell culture and transfection

Human ESGG cell line Eca109 was purchased from the Shanghai Institute of Cell Biology (Shanghai, China). Cells were maintained in RPMI1640 (Invitrogen), supplemented with 10% fetal bovine serum (Invitrogen), 100 U/ml penicillin and 100 μg/ml streptomycin, within a humidified atmosphere containing 5% CO2 at 37°C. 1 × 10^6^ cells cultured in a well of 6-well cell culture plate were transiently transfected. ADH1B-flag of empty vector were generated by retroviral infection. Esophageal squamous cell that constitutively expressed either Flag-tagged ADH1B or empty vector were confirmed by western blot analysis with specific antibodies against ADH1B and the Flag tag.

### Cell proliferation assay and cell cycle

To determine the effect of the polymorphism of ADH1B on proliferation of cancer cell lines, 4 × 10^3^ cells were transfected with ADH1B with mock control following manufacturer’s protocol in 96-well plate. MTT assay was performed at 24 h, 48 h and 72 h post transfection. The absorbance of the samples was measured with spectrophotometer reader at 490 nm. Each assay was performed in triplicate and repeated three times independently.

### MTT assay with alcohol pre-treatment

To determine the effect of ALDH1B on proliferation of Human esophageal squamous cell lines, 1 × 10^5^ cells were transfected with ALDH1B gene (*GG* and *AA*). 2% ethanol was added into the culture medium and negative control group was set at 1%,2%,5%, respectively. MTT assay was performed at 24 h, 48 h and 72 h post transfection. The absorbance of the samples was measured with a spectrophotometer reader at 490 nm. Each assay was performed in triplicate and repeated three times independently.

### Statistical analyses

Differences in the distributions of demographic characteristics, selected variables and genotypes of ADH1B variants between cases and normal controls were evaluated using the χ^2^ test. Associations between ADH1B genotypes and the risk of esophageal cancer were estimated by computing the odds ratios (ORs) and their 95% confidence intervals (CIs) using logistic regression analyses for crude ORs and adjusted ORs when adjusting for age, gender, tobacco use and drinking status. The Hardy-Weinberg equilibrium was tested by a goodness-of-fit χ^2^ test to compare the observed genotype frequencies to the expected among the control subjects. All statistical analyses were conducted using SAS 9.1.3 software (SAS Institute, Cary, NC, USA).

## Results

### Characteristics of the study population

Among the 1001 ESGG cases and the 1391 controls with DNA samples, genotyping was successful in 1001 (100%) cancer case and 1391 (100%) controls for ADH1B rs1229984. Characteristics of cases and controls are summarized in Table 1. Cases and controls appeared to be adequately matched with respect to age and gender as suggested by the x^2^ tests (p = 0.69 and p = 0.46,respectively). No significant difference was observed with regard to drinking status between cases and controls (p = 0.54). However, the prevalence of smoking was higher in the esophageal cancer patients than in the control subjects (P < 0.005) (Table 1).

**Table 1:** General information of esophageal cancer group and control group

	**Esophageal cancer (n = 1001) No. (%)**	**Health control (n = 1391) No. (%)**	P*
Sex			0.69
Male	795 (79.42)	1114 (80.09)	
Female	206 (20.58)	277 (19.91)	
Age (years)			0.46
40	30 (3.00)	47 (3.38)	
41-60	539 (53.84)	778 (55.93)	
>60	432 (43.16)	566 (40.69)	
Smoking status			2.14 × 10^-11^
Non-smoker	337 (33.66)	651 (46.80)	
Smoker	632 (63.14)	684 (49.17)	
Unclear situation	32 (3.20)	56 (4.03)	

*Two-sided χ^2^ test.

### ADH1B rs1229984 polymorphisms and the risk of esophageal cancer

**Figure 1:**
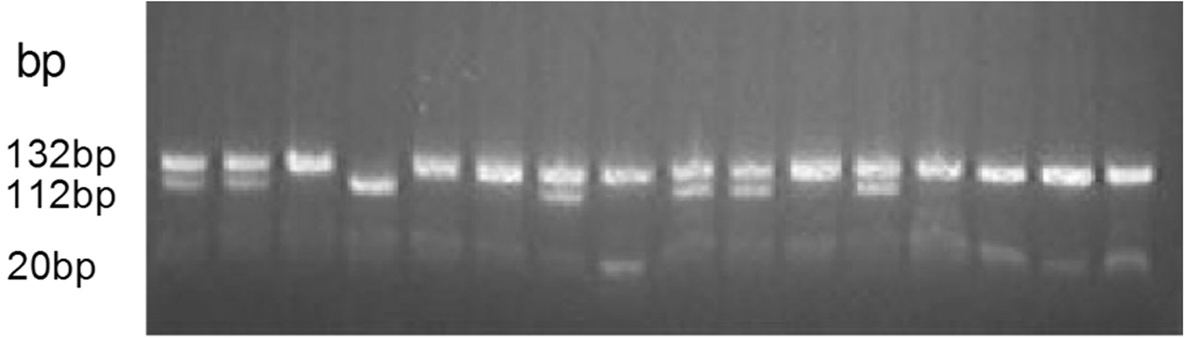
**Electrophoresis of genomic DNA after restriction endonuclease digestion.** ADH1B rs1229984 A/A allele generated one fragment of approximately 132 bp, G/G allele generated two bands of 112-bp and 20-bp, while the lanes containing both132 and 112 fragments represent the allele is A/G.

**Figure 2:**
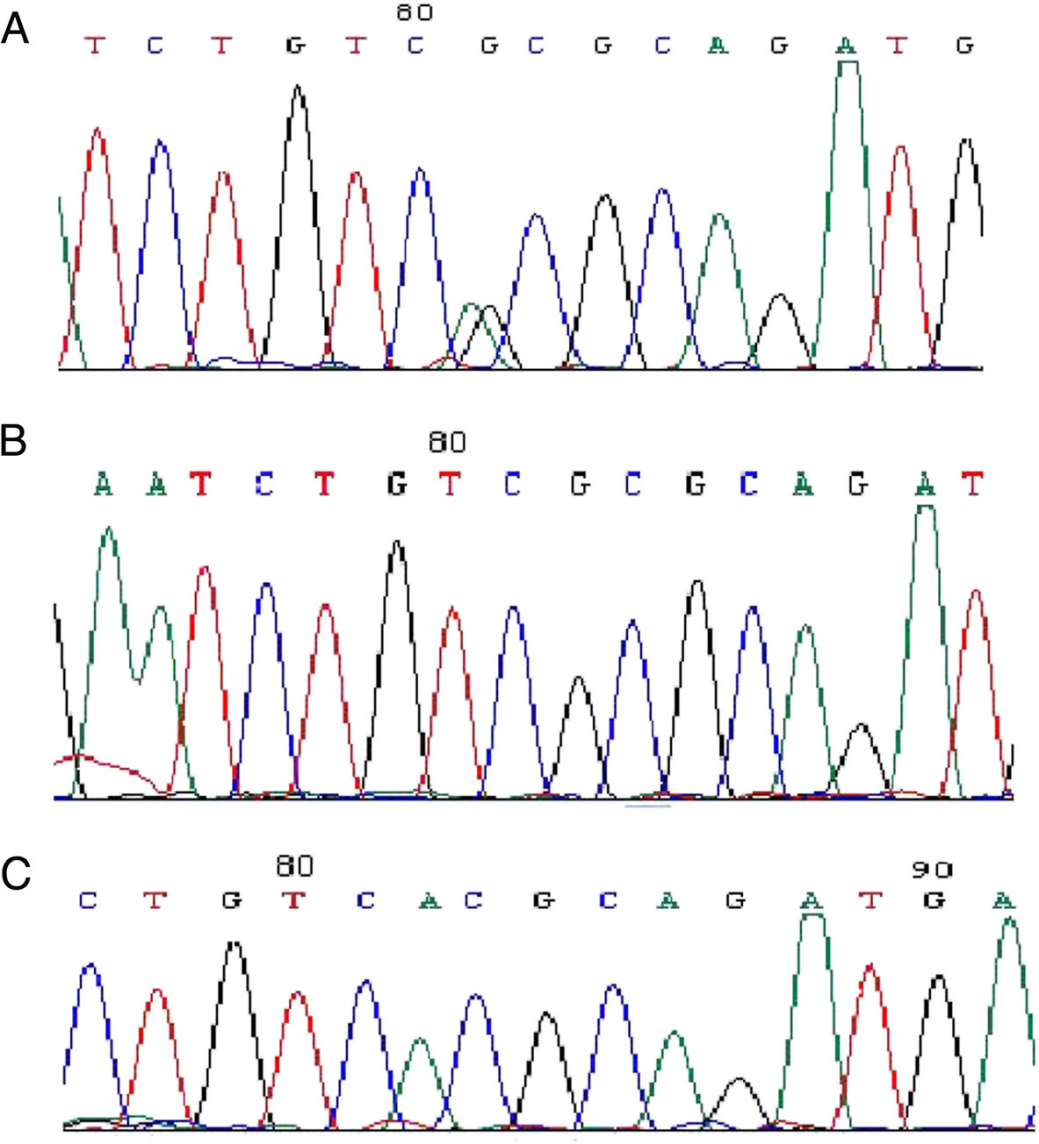
**DNA sequencing of rs1229984 SNP in ESCC patients and healthy controls. A**: genotype A/G; **B**: genotype G/G; **C**: genotype A/A.

Genotyping of ADH1B rs1229984 SNP was successfully sequenced in all subjects. Some of the results from the re-genotyped samples matched that from original ones completely (Figure 1). The distribution of ADH1B rs1229984 SNP (Figure 2) was not correlated with gender and age both in ESCC patients or healthy controls (data not shown). The genotype distribution in control population was in agreement with those expected under Hardy-Weinberg Equilibrium (HWE). The genotype distributions of ADH1B in the cases and controls are shown in Table 2. The observed genotype frequencies for these two polymorphisms in the controls were all within Hardy-Weinberg equilibrium (p = 0.68). In the univariate analyses, the genotype frequencies of ADH1B rs1229984 were 37.66% (AA), 39.96% (AG) and 22.38% (GG) in the patients, and 47.66% (AA), 41.55% (AG) and 10.79% (GG) in the control subjects. The difference was revealed to be significant (p = 0.06). Logistic regression analyses revealed that subjects carrying the GG variant homozygote had a significant 2.81-fold (adjusted OR = 2.81; 95% CI = 2.18-3.62) increased risk of esophageal cancer. In the recessive model, the ADH1B rs1229984 GG variant homozygote was associated with a 1.51-fold significantly increased risk of esophageal cancer compared with rs1229984 AA/AG genotypes (adjusted OR = 1.51, 95% CI = 1.28-1.80).

**Table 2:** Gene frequency of rs1229984 (ADH1B) between esophageal cancer and control group

**Genotype**	**ESGG**		OR* (95% CI)	***P***
	**Patients (n = 1001) No. (%)**	**Controls (n = 1391) No. (%)**		
AA	377 (37.66)	663 (47.66)	1.00 (reference)	
AG	400 (39.96)	578 (41.55)	1.20 (1.00–1.44)	0.06
GG	224 (22.38)	150 (10.79)	2.81 (2.18–3.62)	1.05 × 10^-15^
AG + GG	624 (62.34)	728 (52.34)	1.51 (1.28–1.80)	1.95 × 10^-6^
G allele frequency	42.36%	31.56%		
*P*_trend_^**†**^			<0.01	

*Data were calculated by logistic regression, adjusted for age, sex, smoking.

†Tests for trend of odds within genotypes of AA, AG, GG were two sided and based on likelihood ratio tests assuming a multiplicative model.

### Enzyme activity

**Figure 3:**
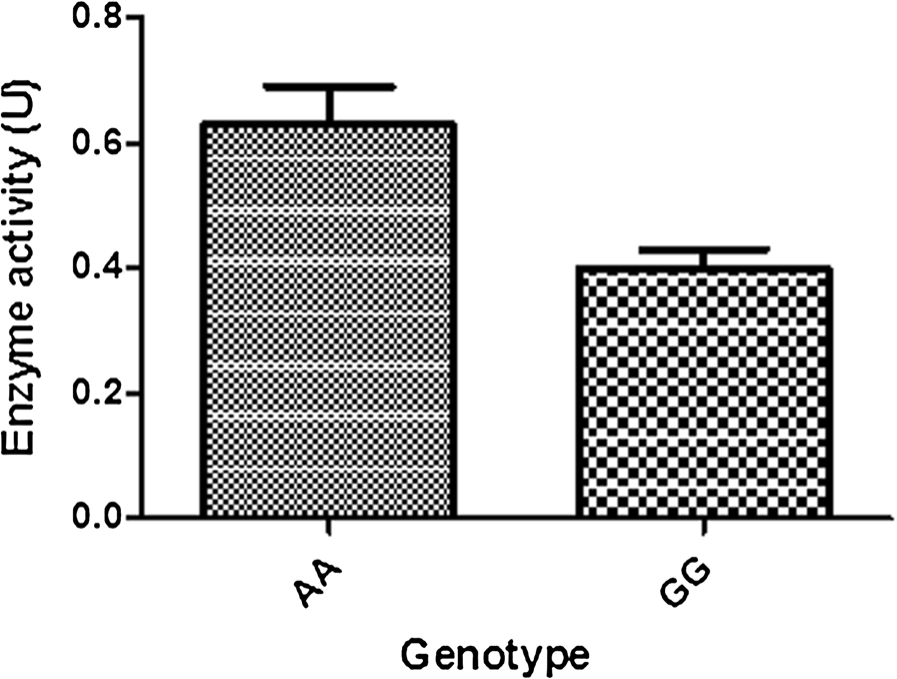
Enzyme activity of different genotype (*AA *and *GG*). * represent statistical significance with p _value_ < 0.05.

**Figure 4:**
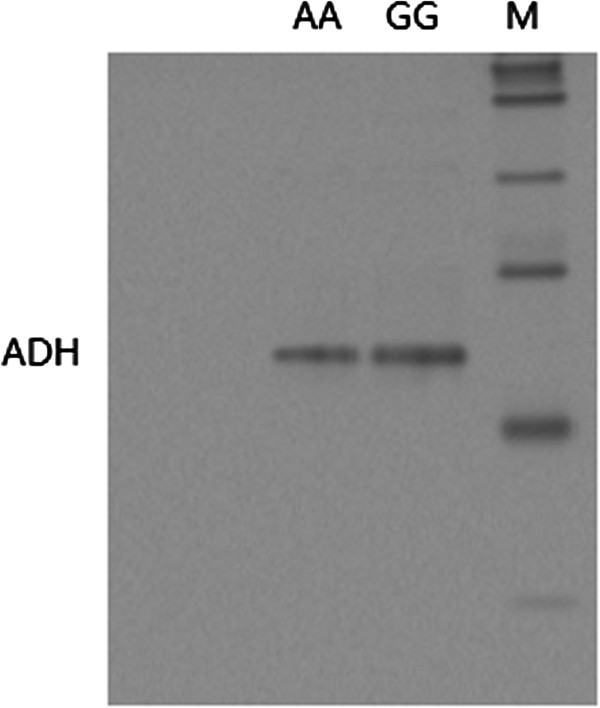
**Quantitative expression of ADH in genotype *AA *** **and *GG*.**

One unit of enzyme activity was defined as the amount which produced 1uM of NADH per min. The NADH activity was assayed by monitoring the increase in absorbance at 340 nm due to the formation of NADH. The enzyme was ADH1B activity can be varied according to the Polymorphism of the rs1229984. The formaldehyde accumulated during Enzyme activity dropped as we can see from the Figure 3 and Figure 4.

### ADH1B activity promote cell proliferation

**Figure 5:**
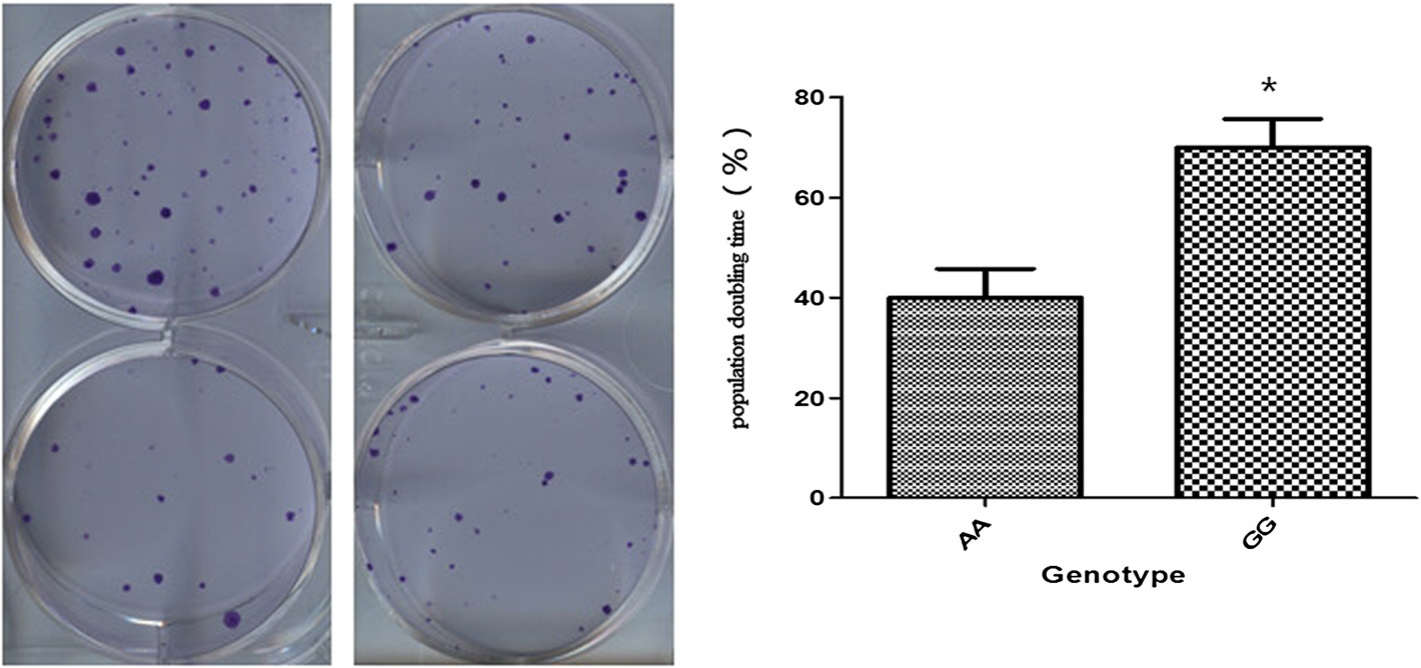
ADH1B activity promote cell proliferations (*AA *and *GG*). *represent statistical significance with p _value_ < 0.05.

To explore the role of ADH1B enzyme activity on the proliferation of esophageal cancer cell line with Exogenous of alcohol. The tolerance of cells with ADH1B rs1229984 AA genotype was better than the rs1229984 GG genotype. The Alcohol Pre-treatment experiment confirmed that the genotype of ADH1B rs1229984 was significantly associated with the tolerance to alcohol Pre-treatment. Collectedly, these results indicate that ADH1B may play a role in the proliferation of esophageal cancer cell line (Figure 5).

## Discussion

We investigated the associated of ADH1B SNPs with risk of esophageal cancer in a high-risk Chinese population. Multivariable logistic analysis revealed that the ADH1B rs1229984 GG genotype was associated with an increased risk of esophageal cancer, and that this effect was more evident among males, younger subjects and smokers. Our results suggested a potential role of ADH1B SNPs on the etiology of esophageal cancer.

Two recent genome-wide association studies identified the variation of ADH1B rs1229984 as risk factors for esophageal cancer in a Japanese population. However, in another three genome-wide association studies in larger Chinese populations, the results presented negative or protective efforts of these polymorphisms for esophageal cancer risk [17]. The reason for these inconsistent findings for ADH1B rs1229984 polymorphisms is unknown. However, variation in enzyme activity with ethnicity and gender could contribute to differences in influences on neoplasms. The genome instability induced by ethanol and acetaldehyde mediated pathways could explain ADH1B polymorphic effects on alcohol induced carcinogenesis. In the present study, we also found a significant gene environment interaction between ADH1B rs1229984 polymorphisms and smoking habit, suggesting susceptibility to esophageal cancer.

Alcoholic beverages may have carcinogenic effects on humans and are causally related to cancer of the oral cavity, pharynx, larynx and esophagus. The genetic polymorphisms of alcohol-metabolizing enzymes modulate individual differences in alcohol-oxidizing capability and drinking behavior. Genetic variants such as alcohol dehydrogenases (ADH) relates to average alcohol consumption or circulating ethanol levels deserve our attention. ADHs catalyze the oxidation of alcohols to aldehydes, which are low Km (Michaelis constant)-class enzyme [18,19]. There are five ADH classes existing in humans [20] and functional polymorphisms of ADH1B and ADH1C genes produce iso-enzymes with different maximal activities (V_max_) and affinities for ethanol. One of the coding variant in ADH1B is rs1229984, which leads to the replacement of Arg48 with His48, is common in Asian populations and the enzymes with His48 oxidize ethanol approximately 70- to 80-fold faster than those with Arg48, eventually reduces their risk for alcoholism [21]. These results are consistent with the recent analysis showed that AG (Arg/His) or AA (His/His) phenotype exhibited higher activity compared to GG (Arg/Arg).

To investigate the function connection of ADH1B in the regulation of ESCC cell proliferation, we further evaluate the proliferation capacity of cells transfected with ADH1B (*GG* or *AA*) plasmid. Notably, cells contrasfected with ADH1B (*GG*) had a significantly higher proliferative index than those transfected with ADH1B (*CC*) plasmid. Meanwhile, cells transfected with ADH1B (*GG*) exhibited a significantly higher colony forming efficiency, as well as a significantly shorter population doubling time than the ADH1B (*CC*) cells (Figure 5). This result, combined with the fact that the enzyme activity of ADH1B with ADH1B (*CC*) in the cells was significantly higher than that ADH1B (*GG*) (Figure 3 and Figure 4), suggest that ADH1B might regulate the proliferation of ESCC cells associated with enzyme activity.

In conclusion, the present study provided marked evidence that functional polymorphism of ADH1B rs1229984 may contribute to the risk of esophageal cancer. However, our results were obtained with a limited sample size and therefore only preliminary conclusions can be drawn. Besides, the underlying mechanisms of ADH1B in regulating the proliferation of ESCC cell warrant further investigation.

## Competing interests

The authors declare that they have no competing interests.

## Authors’ contributions

BY and CYJ participated in the design of this study, and they both performed the statistical analysis. YZ carried out the study, together with WL, collected important background information, and drafted the manuscript. JF and XZ conceived of this study, and participated in the design and helped to draft the manuscript. All authors read and approved the final manuscript.

## Authors’ informations

Bo Ye and Chun-Yu Ji should be regarded as co-first authors.

## References

[B1] Ke L Mortality and incidence trends from esophagus cancer in selected geographic areas of China circa 1970–90 Int J Cancer 2002 102 3 271 274 12397650 10.1002/ijc.10706

[B2] Chen J Zhang N Ling Y Wakai T He Y Wei L Wang S Akazawa K Alcohol consumption as a risk factor for esophageal adenocarcinoma in North China Tohoku J Exp Med 2011 224 1 21 27 21505271 10.1620/tjem.224.21

[B3] Holmes RS Vaughan TL Epidemiology and pathogenesis of esophageal cancer Semin Radiat Oncol 2007 17 1 2 9 17185192 10.1016/j.semradonc.2006.09.003

[B4] Montesano R Holestein M Hainaui P Genetic alterations in esophageal cancer and their relevance to etiology and pathogenesis: a review Int J Cancer 1996 69 3 225 235 8682592 10.1002/(SICI)1097-0215(19960621)69:3<225::AID-IJC13>3.0.CO;2-6

[B5] Blot WJ McLaughlin JK The changing epidemiology of esophageal cancer Semin Oncol 1999 26 2 8 10566604

[B6] Hardikar S Onstad L Blount PL Odze RD Reid BJ Vaughan TL The role of tobacco, alcohol, and obesity in neoplastic progression to esophageal adenocarcinoma: a prospective study of Barrett’s Esophagus PLoS One 2013 8 1 e52192 23300966 10.1371/journal.pone.0052192 PMC3536789

[B7] Blot WJ Alcohol and cancer Cancer Res 1992 52 7 Supplement 2119s 2123s 1544150

[B8] Agarwal D Genetic polymorphisms of alcohol metabolizing enzymes Pathol Biol 2001 49 9 703 709 11762132 10.1016/s0369-8114(01)00242-5

[B9] Yang C Matsuo K Ito H Hirose K Wakai K Saito T Shinoda M Hatooka S Mizutani K Tajima K Esophageal cancer risk by ALDH2 and ADH2 polymorphisms and alcohol consumption: exploration of gene-environment and gene-gene interactions Asian Pac J Cancer P 2005 6 3 256 16235983

[B10] Asakage T Yokoyama A Haneda T Yamazaki M Muto M Yokoyama T Kato H Igaki H Tsujinaka T Kumagai Y Genetic polymorphisms of alcohol and aldehyde dehydrogenases, and drinking, smoking and diet in Japanese men with oral and pharyngeal squamous cell carcinoma Carcinogenesis 2006 28 4 865 874 17071628 10.1093/carcin/bgl206

[B11] Tóth R The effect of alcohol dehydrogenase gene polymorphisms on alcohol consumption and chronic liver diseases in Hungary 2011

[B12] Eng MY Luczak SE Wall TL ALDH2, ADH1B, and ADH1C genotypes in Asians: a literature review Alcohol Res Health 2007 30 1 22 17718397 PMC3860439

[B13] Gu H Gong D Ding G Zhang W Liu C Jiang P Chen S Chen Y A variant allele of ADH1B and ALDH2, is associated with the risk of esophageal cancer Exp Ther Med 2012 4 1 135 140 23060937 10.3892/etm.2012.547 PMC3460276

[B14] Wang L-D Zhou F-Y Li X-M Sun L-D Song X Jin Y Li J-M Kong G-Q Qi H Cui J Genome-wide association study of esophageal squamous cell carcinoma in Chinese subjects identifies susceptibility loci at PLCE1 and C20orf54 Nat Genet 2010 42 9 759 763 20729853 10.1038/ng.648

[B15] Tanaka F Yamamoto K Suzuki S Inoue H Tsurumaru M Kajiyama Y Kato H Igaki H Furuta K Fujita H Strong interaction between the effects of alcohol consumption and smoking on oesophageal squamous cell carcinoma among individuals with ADH1B and/or ALDH2 risk alleles Gut 2010 59 11 1457 1464 20833657 10.1136/gut.2009.205724

[B16] Quayle J Taylor G Carbon assimilation by Pseudomonas oxalaticus (OX1). 5. Purification and properties of glyoxylic dehydrogenase Biochem J 1961 78 3 611 13738657 10.1042/bj0780611 PMC1205383

[B17] Cui R Kamatani Y Takahashi A Usami M Hosono N Kawaguchi T Tsunoda T Kamatani N Kubo M Nakamura Y Functional variants in < i > ADH1B</i > and < i > ALDH2</i > coupled with alcohol and smoking synergistically enhance esophageal cancer risk Gastroenterology 2009 137 5 1768 1775 19698717 10.1053/j.gastro.2009.07.070

[B18] Mardh G Vallee BL Human class I alcohol dehydrogenases catalyze the interconversion of alcohols and aldehydes in the metabolism of dopamine Biochemistry 1986 25 23 7279 7282 2432930 10.1021/bi00371a005

[B19] Kayaalti Z Soylemezoglu T Distribution of ADH1B, ALDH2, CYP2E1 *6, and CYP2E1 *7B genotypes in Turkish population Alcohol 2010 44 5 415 423 20598484 10.1016/j.alcohol.2010.06.002

[B20] Matsushima T [ADH isoenzymes] Nihon Rinsho 1995 53 5 1237 1240 7602785

[B21] Bierut LJ Goate AM Breslau N Johnson EO Bertelsen S Fox L Agrawal A Bucholz KK Grucza R Hesselbrock V ADH1B is associated with alcohol dependence and alcohol consumption in populations of European and African ancestry Mol Psychiatr 2012 17 4 445 450 10.1038/mp.2011.124 PMC3252425 21968928

